# Effectiveness of SBAR-based simulation programs for nursing students: a systematic review

**DOI:** 10.1186/s12909-023-04495-8

**Published:** 2023-07-14

**Authors:** Jungmi Yun, Yun Ji Lee, Kyoungrim Kang, Jongmin Park

**Affiliations:** College of Nursing, Research Institute of Nursing Science, Pusan National University, Yangsan, 50612 Republic of Korea

**Keywords:** Communication, Simulation training, Nursing education, Systematic review

## Abstract

**Background:**

Situation, background, assessment, and recommendation (SBAR) has been extensively used in clinical and nursing education. A structured communication program increases effective communication, positivity, and education satisfaction during inter-professional collaboration among nursing students. This systematic review aimed to identify and synthesize evidence on the effectiveness of SBAR-based simulation training for nursing students.

**Methods:**

A research protocol was developed according to the Preferred Reporting Items for Systematic Review and Meta-Analysis Protocols guidelines. The protocol for this study was registered in PROSPERO (CRD42021234068). Eight bibliographical databases were searched for studies published between 2001 and 2021, using relevant search terms. Searches were conducted in PubMed, Embase, Cumulative Index to Nursing and Allied Health, and Cochrane Central Register of Controlled Trials for literature in English, and DBpia, Research Information Sharing Service, Korean Studies Information Service System, and Korea Institute of Science and Technology Information for literature in Korean. After screening titles, abstracts, and full-text papers, pertinent data were extracted, and critical appraisals of the retrieved studies were performed. Data were analyzed using the framework approach, and the findings were presented in a narrative summary. The Effective Public Health Practice Project “Quality Assessment Tool for Quantitative Studies” was used to assess the quality of the included studies.

**Results:**

Twelve studies were included: 3 randomized controlled trials and 9 quasi-experimental studies. Two overarching themes were noted, namely communication clarity and critical thinking. The results of six out of 12 studies produced significant results in favor of SBAR-based simulation in terms of communication clarity. Divergent results were obtained regarding communication ability, critical thinking, confidence, learning self-efficacy, and attitude toward patient safety. The results of these studies highlight that communication clarity ultimately leads to positive results in terms of nursing students’ behaviors related to patient safety.

**Conclusions:**

This review provides a comprehensive update of the literature on the effectiveness of SBAR-based nursing simulation programs for nursing students. These programs were found to have positive learning outcomes because of clear and concise communication. Further studies on the effectiveness of various learning outcomes derived from SBAR-based programs are required.

## Background

Accurate communication skills among healthcare professionals are very important in the current healthcare environment, where multidisciplinary care and collaborative practice are recommended. A nurse’s ability to communicate is one of the most important competencies for efficiently providing information necessary to report a patient’s condition. A nurse’s clear communication ability contributes to improving the quality of nursing and minimizing accidents that may occur in clinical settings [1, 2].

Situation, background, assessment, and recommendation (SBAR) has been extensively used in clinical and healthcare educational settings [3]. The SBAR includes the communication of the patient’s current situation, the background and causes of the situation, the assessment of the current condition, and the reporter’s recommendations for further treatment [3]. SBAR is a reliable and validated communication tool that can be easily implemented in hospital-based practices for sharing information among healthcare providers [4] and is a structured communication tool that enables clear communication in a short time [5].

Nursing students are expected to develop practical nursing competencies and communication skills through theoretical learning and clinical practice [6]. Still, many nursing college students merely observe in their clinical training. That is, their attitudes are pretty passive, which makes it challenging to achieve these educational goals. Simulation-based education may be a helpful supplement in clinical practice for nursing students to address this issue. This can improve nursing competencies by enabling iterative and direct learning using virtual scenarios [7].

A structured communication program increases effective communication, positivity, and education satisfaction during inter-professional collaboration among nursing students [3]. In previous studies, incorporating SBAR techniques into simulation-based education positively affected communication skills, clarity, and confidence [8–10]. Using SBAR, nurses can more accurately recognize patient condition changes, enabling precise, effective, enhanced communication and cooperation among healthcare staff [11, 12]. Research on the effectiveness of SBAR in nursing education is still ongoing, and it is necessary to promote its implementation in the curriculum sufficiently.

As a result of reviewing research on structured communication programs in Korea, studies such as the SBAR program have been conducted using a combination of theory lectures, role-play, discussion, debriefing, team activity, case-based, and simulation methods [1, 13]. Many overseas studies have applied a communication promotion program to nursing education using theoretical lectures, role-play, theater therapy techniques, online media use, simulations, pamphlets, reflection, feedback and discussion, and DVD viewing [14–17].

Most communication programs implemented for nurses or nursing students had statistically significant effects. Still, the concept and evidence of the program were not uniform, and the tools used by each researcher, research participants, and measurement period varied. Although simulation education is becoming more important in clinical practice when a simulation program using SBAR is applied, contradictory results (effective/ineffective) have been reported as research results, and the lack of high-quality literature (low-modest) was confirmed. Each program has a different composition, contents, and results; therefore, it is necessary to systematically examine the contents and effects of various simulation programs using SBAR [18]. Accordingly, the contents, effects, and trends of the SBAR-based programs were comprehensively reviewed and integrated to provide the best basis for future communication program development for nursing students. This systematic review aimed to identify and synthesize evidence on the effectiveness of SBAR-based simulation programs for nursing students.

## Methods

This systematic review aimed to integrate and analyze the effects of SBAR-based nursing simulation programs for nursing students. The primary research question guiding this systematic review is: What is the impact of the SBAR-based simulation program on nursing students? To address this question, we followed the Preferred Reporting Items for Systematic Reviews and Meta-Analyses (PRISMA) guidelines for systematic reviews and meta-analyses [19]. The study protocol was registered on the PROSPERO website (CRD42021234068; https://www.crd.york.ac.uk/PROSPERO/).

## Eligibility criteria

This study applied the PICO-SD (participants, intervention, comparison, outcomes, study design) tool as follows: (1) participants (P): nursing students; (2) intervention (I): nursing simulation programs that utilized SBAR-centered scenarios or activities; (3) comparison (C): different simulation programs or other educational interventions; (4) outcome (O): significant effects of the intervention; and (5) study design (SD): randomized controlled trial (RCT), or quasi-experimental design. Studies with nursing students as participants, either exclusively or as part of a sample including other healthcare students/professionals, are eligible for inclusion. The following studies were excluded: (1) single-arm studies, (2) observational studies, qualitative studies, mixed method studies, review articles, editorials, case studies, and proceedings, and (3) pilot studies. The publication year of the articles was limited from January 1, 2001, to June 30, 2021.

## Search strategies and study selection

A systematic literature review was conducted for articles published from January 1, 2001, to June 30, 2021. We searched international studies in the following databases: PubMed, Embase, Cochrane Central Register of Controlled Trials (CENTRAL), and Cumulative Index to Nursing and Allied Health (CINAHL). Domestic studies were searched in DBpia, Research Information Sharing Service (RISS), Korean Studies Information Service System (KISS), and Korea Institute of Science and Technology Information (Kisti). The keyword selection and search included Medical Subject Headings (MeSH) and Emtree for thesaurus in biomedical and life sciences. The keywords included “nursing,” “SBAR,” “ISBAR,” “SBAR-R,” “simulation,” “program*,” and “intervention*.” The search was limited to articles written in Korean or English.

In title screening, two independent reviewers (J.Y. and J.P.) examined the identified records’ titles to exclude irrelevant studies. Any discrepancies were resolved through discussion or consultation with a third reviewer (K.K.), if necessary. Next, the two reviewers examined the abstracts and keywords of the remaining records to refine the list of potentially relevant studies further. Any disagreements at this stage were also addressed through discussion or consultation with the third reviewer. Finally, the two reviewers independently assessed the full-text articles of the remaining studies for eligibility according to the pre-specified inclusion and exclusion criteria. Disagreements at this stage were also resolved through discussion or consultation with a third reviewer. We also manually screened the reference lists of the included studies and relevant reviews to ensure that all pertinent studies were identified.

## Data extraction

To ensure the objectivity of data extraction, two reviewers (K.K. and Y.L.) independently extracted data from the included studies. We collected data regarding the authors, year of publication, country, study design, subjects, sample size, intervention characteristics, control groups, and outcome measurements. In case of disagreement between researchers, a consensus was reached by discussion with a third reviewer (J.Y.).

## Quality assessment

We used the Effective Public Health Practice Project (EPHPP) “Quality Assessment Tool for Quantitative Studies” [20] to assess the quality of the included studies. Reviewers provided strong, moderate, or weak ratings for the following domains: selection bias, design, confounders, blinding, data collection methods, and withdrawals and dropouts. Strong, moderate, and weak global ratings were determined according to the number of weak ratings received [20]. The EPHPP tool was used to assess the quality of both RCT and quasi-experimental studies included in our systematic review. While the tool is applicable to both study designs, slight modifications were made as needed to accommodate the differences between RCTs and quasi-experimental studies. Two independent authors (J.Y. and J.P.) assessed the quality of the included studies, and any disagreements were resolved through discussion or consultation with a third reviewer (K.K.), if necessary.

## Results

### Search results

**Fig. 1:**
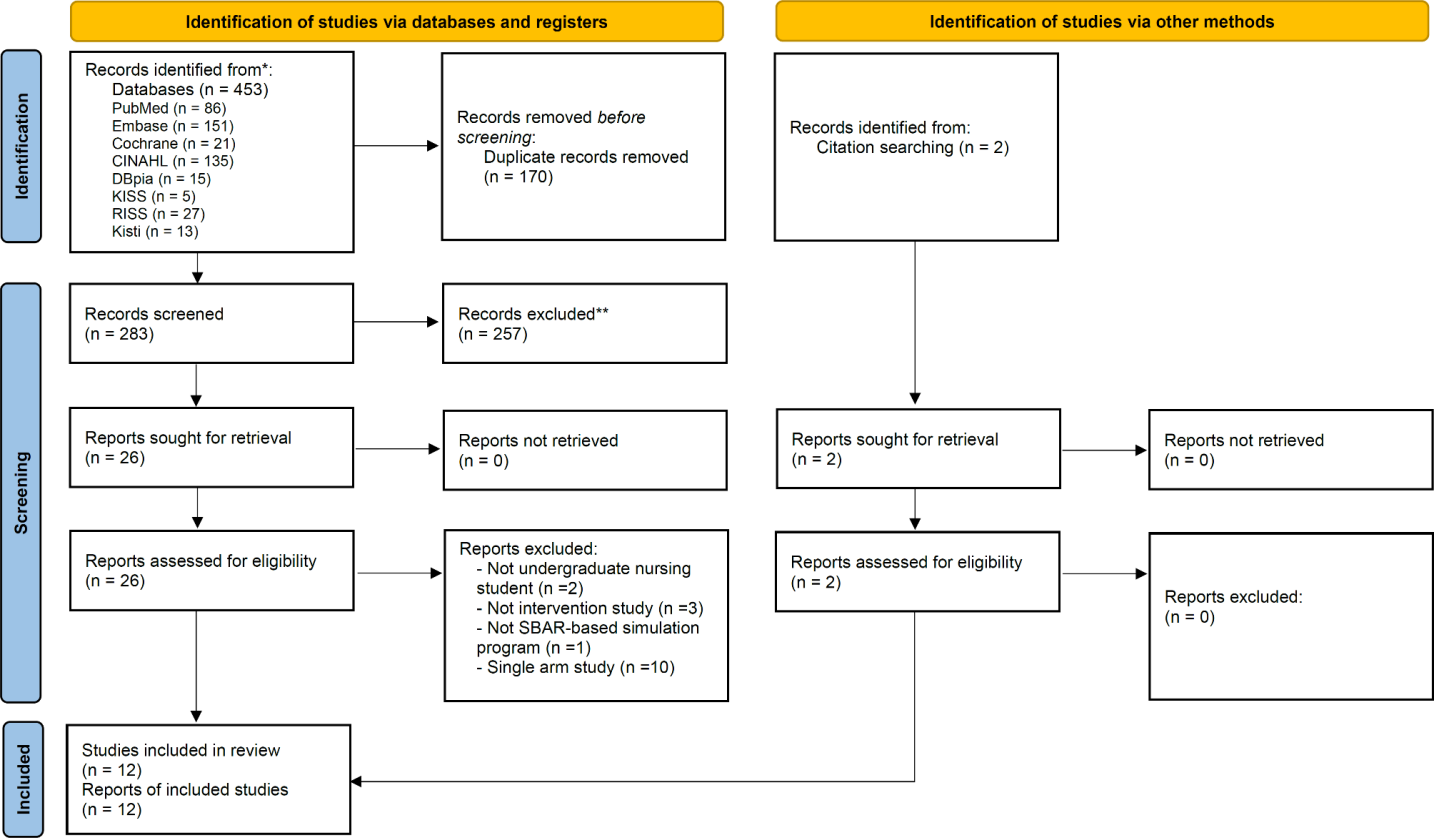
PRISMA flow diagram of the study selection process

Figure 1 shows the flow of the study selection process for this review. After searching eight databases, 453 studies were found. A total of 170 studies were removed as duplicates and the titles and abstracts of 283 studies were screened. Due to irrelevancy, 257 studies were excluded, the full texts of 26 studies were reviewed, and two additional articles were searched and reviewed from other sources. Ultimately, 12 studies were included in the narrative analysis.

### Description of the included studies

Table 1 presents the characteristics of the studies included in the narrative analysis. Of the 12 included studies, most were conducted in Korea [3, 21–28] and one each in Ireland [29], Spain [30], and the USA [31]. A quasi-experimental design was used in nine studies [3, 21, 23–28, 31] and a RCT was adopted in the remaining three studies [22, 29, 30]. In two studies [24, 29], more than one experimental group was designated. The total sample size was 886 participants (503 in the intervention group and 383 in the control group). The individual sample size of each group— the experimental and control groups of the included studies—was mostly under 50, and the average of both experimental and control groups was approximately 34.7.

**Table 1:** Characteristics of Included Studies

Author (year) /Country	Design	Participants /Sample size	Intervention	Control	Outcomes	Global rating on quality appraisal
Breen et al. (2019) / Ireland	RCT	Nursing and Medical students E1: 23(N:12/M:11) E2: 25(N:14/M:11) C: 29(N:14/M:15)	E1: E-learning + Standard simulation - SBAR call training (210 min) - Practice four cases with partner - Performance one case with facilitator E2: E-learning + proficiency-based progression - SBAR call training (210 min) - Practice four cases with partner - Performance one case with facilitator	National e-learning program only	Demonstrated proficiency: - C vs. E1 (χ ^2^ =22.25, p < .001) - E1 vs. E2 (χ ^2^ =11.04, p = .001) Completed step: - C vs. E1 (p < .001) - E1 vs. E2 (p < .001) Errors and critical errors: - C vs. E1 (p < .001) - E1 vs. E2 (p = .010)	Weak
Chae, (2019) / Korea	Quasi-experimental	Nursing students E: 30/C: 30	Education, Practice and Simulation - SBAR lecture (60 min) - SBAR practice with videos and role-play (120 min)	Self-learning (1 h) Group discussion and presentation on patient safety (2 h)	Communication clarity (t=-2.49, p = .020), Self-leadership (t = 11.75, p = .001) Attitude toward patient safety (t = 5.12, p = .027) Safety care performance (t = 7.85, p = .007)	Weak
Jeong and Kim (2020) / Korea	RCT	Nursing students E: 26/C: 28	SBAR-based fall simulation program - Orientation (120 min) - SBAR skill training with role-play (100 min) - Evaluation and discussion (25 min)	The general handoff-based fall simulation program	Communication clarity (t = −11.28, p < .001) Fall-related patient skill (F = 11.71, p= .001)	Moderate
Lee (2021) / Korea	Quasi-experimental	Nursing students E: 48/C: 48	SBAR education before CP SBAR role-play four times during CP (120 min)	Regular CP	Communication competence (p< .001) Critical thinking (p < .001)	Weak
Noh and Kim (2021) / Korea	Quasi-experimental	Nursing students E1: 31 E2: 29 E3: 33	E1: Assertiveness training - Orientation (10 min) and education (20 min) - Practice of assertiveness skills (20 min) - Group discussion (10 min) E2: SBAR training - Orientation (10 min) and education (20 min) - SBAR role-play (20 min) - Group discussion (10 min) E3: Assertiveness + SBAR training - Orientation (5 min) - Assertiveness training (education 10 min + practice 15 min) - SBAR training (education 15 min + role-play 15 min) - Group discussion (5 min)		Communication clarity (p = .006) Clinical practice stress (p = .001) Clinical competence (p = .023)	Moderate
Noh et al. (2016) / Korea	Quasi-experimental	Nursing students E: 56/C: 49	- Pre-briefing (30 min) + SBAR education (60 min) - SBAR scenario performance (70 min) - SBAR nursing report (20 min) + debriefing (30 min)	Pre-briefing (30 min) + scenario performance (70 min) + general nursing report (20 min) + debriefing & SBAR education (60 min)	Report clarity Situation 1 (χ ^2^ =26.30, p < .001), Background 1 (χ ^2^ = 20.67, p < .001), Background 2 (χ ^2^ =30.62, p < .001), Recommendation 1 (χ ^2^ = 36.82, p < .001) Recommendation 2 (χ ^2^ =47.20, p < .001) Confidence (t = 2.75, p = .007)	Weak
Raurell-Torredà et al. (2021) / Spain	RCT	Nursing students E: 48/C: 45	- Pre-briefing + self-evaluation + instruction during simulation - Role-play (60 min)	Same intervention excluding role-play	Patient safety (p = .015) Patient assessment (not significant) Patient intervention (not significant) Critical thinking (not significant)	Moderate
Seong and Yoon (2018) / Korea	Quasi-experimental	Nursing students E: 28/C: 25	- Lecture (120 min) - Practice with SBAR simulation: pre-briefing, scenario simulation, and debriefing (240 min)	Conventional learning: lectures, demonstrations and individual practice	Critical thinking (F = 6.28, p = .015) Self-efficacy (F = 15.63, p = .001)	Weak
Uhm et al. (2019) / Korea	Quasi-experimental	Nursing students E: 40/C: 41	CP + SBAR training course (240 min) − 4 phase: concrete experience, reflective observation, abstract conceptualization, and active experimentation - Using orientation, role-play, observing, feedback, small group teaching, and discussion	Learned regular nursing processes and therapeutic communication	SBAR communication (F = − 11.18 p < .001) Communication clarity (t = − 12.11 p < .001) Handover confidence (t = − 4.40 p < .001)	Weak
Yeh et al. (2019) / USA	Quasi-experimental	Nursing students E: 22/C: 21	Asynchronous online deliberate practice (DP) sessions (45 min) - Record SBAR report, feedback, reflection, repeat practice, and feedback - Baseline practice (DP1) + evaluation practice (DP5) - Completed others DP sessions and reported real-life practice experience (DP2-4)	- Baseline practice (DP1) + evaluation practice (DP5) - Brief experience survey to report any real-life practice	Performance (p = .010, 95% CI = 0.25–1.81) Confidence (p = .020, 95% CI = 0.14–1.76)	Weak
Yoon and Lee (2018) / Korea	Quasi-experimental	Nursing students E: 33/C: 36	SBAR team-based simulation program (6 h for 2 weeks) - Orientation before class - Readiness test + Q&A (1st week) - Case study + Presentation using conceptual diagram + Introduction SBAR + Q&A (2nd week)	Conventional learning: lectures, demonstrations and individual practice	Critical thinking (F: 11.91, p < .001) Clarity of communication (F = 4.40, p = .040)	Weak
Yu and Kang (2017) / Korea	Quasi-experimental	Nursing students E: 31/C: 31	- Lecture (60 min) - Role-play on four sessions (each 120 min) - Critical thinking & Team-based simulation learning (120 min)	Lecture + CP	Communication clarity (t = − 5.50, p< .001) SBAR communication (t = − 3.05, p= .003) Satisfaction (t = − 4.94, p< .001) Confidence (not significant)	Weak

Abbreviations: C, control group; CI, confidence interval; CP, clinical practicum; N, nursing students; M, medical students; Q&A, questions and answers; SBAR, situation-background-assessment-recommendation; RCT, randomized controlled trial; E, experimental group.

### Interventions of the included studies

The interventions in the included studies varied in detail, including orientation, pre-briefing, and role-play. The duration of the programs also varied between one and six hours. Except for one study [3], that applied a four-phase clinical practicum (CP) with SBAR training, the other eight studies in Korea included orientation, lecture, or educational sessions for more than 60 min before the simulation scenario performance with role-play. A recent study [24] applied for a three-session program. Each session consisted of education with orientation for 20 min, assertiveness skills or role-play for 15–20 min, and group discussion for 10 min. Other studies mostly ran a program comprising education with 60–120 min of orientation, role-play for 60–120 min, and debriefing/discussion for about 30 min. One study [29] adopted e-learning with simulations for a two-session program. Compared to the experimental groups, programs applied to the control groups included a diverse range of program in nursing education studies. These included e-learning programs, self-learning, group discussions, simulation programs, regular clinical practice, pre-briefing and debriefing sessions, conventional learning methods, and a focus on nursing processes and therapeutic communication. The variety of programs provided a comprehensive understanding of different approaches in nursing education and allowed for thorough evaluation of the experimental group interventions.

### Outcomes of the included studies

Six studies measured “communication clarity” using communication clarity scale (CCS) by Marshall et al. [32], and one study [22] used a structured communication tool [33]. The “communication ability” was evaluated using the 15-item general interpersonal communication competence scale (GICC) [34] in three studies [22–24]. Other communication-related variables were “report clarity”, measured by two items each for SBAR [25], “capacities to identify roles and to communicate”, measured by KidSIM team performance (KidSIM-TPS) [30], “SBAR communication accuracy”, using the tool developed by Yu & Kang [3], and “SBAR communication” itself by checklist [27]. The other outcomes for examining the effects of the interventions included “confidence”, scored on visual analogue scale (VAS) in four studies [3, 25, 27, 31], “clinical competence”, scored by the clinical competence instrument by Lee in one study [24], “self-efficacy” by general self-efficacy scale [28] and learning self-efficacy scale [3], and “critical thinking”, rated with the critical thinking instrument by Yoon in three studies [23, 26, 28].

### Quality of included studies

An overview of the quality of the included studies is shown in Table 2 and the global ratings are presented in Table 1. Of the 12 studies included in the present systematic review, nine studies were classified as weak, three as moderate [22, 24, 30], and none were classified as strong. Most of the studies were appraised as weak at the “selection bias” (11 out of 12 studies) and “blinding” (nine studies), whereas 11 studies were rated as strong at “confounders” and “withdrawals and dropouts”. Eight studies were evaluated as strong in the “data collection method” category. In the “study design” section, 10 studies were classified as moderate in consideration of randomization.

**Table 2:** Quality Assessment of Included Studies Using the EPHPP Tool

Component of Ratings	Breen et al. (2019)	Chae (2019)	Jeong and Kim (2020)	Lee (2021)	Noh and Kim (2021)	Noh et al. (2016)	Raurell-Torredà et al. (2021)	Seong and Yoon (2018)	Uhm et al. (2019)	Yeh et al. (2019)	Yoon and Lee (2018)	Yu and Kang (2017)
**Selection Bias**	W	W	W	W	M	W	W	W	W	W	W	W
**Study Design**	S	M	M	M	M	M	S	M	M	M	M	M
**Confounders**	S	S	S	S	S	S	S	S	S	W	S	S
**Blinding**	W	W	M	W	W	W	M	W	W	S	W	W
**Data Collection Method**	W	S	S	S	S	W	M	S	S	W	S	S
**Withdrawals and Dropouts**	S	S	M	S	S	S	S	S	S	S	S	S
**Global Rating**	W	W	M	W	M	W	M	W	W	W	W	W

Abbreviations: EPHPP, Effective Public Health Practice Project; S, strong; M, moderate; W, weak

## Discussion

### Overview of findings

The objective of this systematic review is to examine and synthesize the available evidence regarding the effectiveness of SBAR-based simulation training for nursing students. In this discussion, we will address two main themes: communication clarity and beyond communication, which encompasses communication ability, critical thinking, self-leadership, patient safety, confidence, and self-efficacy. Our findings suggest that SBAR-based simulation programs have the potential to enhance nursing students’ communication clarity, thereby contributing to improved communication in clinical settings.

### Impacts of interventions

Six of the included studies measured the clarity of communication. A previous study showed that teaching SBAR techniques to healthcare providers can improve communication clarity in both classroom and clinical settings [35]. Adaptation to clinical practice is significant for novice nurses entering the clinical environment after graduation [27]. As an approach to address the communication difficulties of new nurses in the early stages of adjustment, offering a program including SBAR before graduation improved communication and information organization skills and increased the reliability of information transmission [36]. Previous studies have measured fidelity to SBAR by determining the extent to which users perform SBAR as intended (e.g., measures of adherence to the mnemonic during communication). Classroom-based studies achieved levels of fidelity to SBAR ranging from 71–87% and reported moderate to considerable improvements in the clarity of communication [3, 32, 37].

On the other hand, studies conducted in clinical settings have shown no or only moderate improvements in clarity, with fidelity ranging from 53–83% [38–40]. The lesser improvements in communication clarity seen in studies from clinical settings suggest the need to establish higher fidelity to SBAR as intended [35]. In other words, implementing without confirming adherence or exposing nursing students to SBAR only in classroom settings does not lead to the planned improvement in communication. Therefore, preparing a method to check and monitor fidelity to SBAR in a simulation program that reproduces the clinical situation is necessary.

In addition to communication ability, critical thinking, self-leadership, patient safety, confidence, and self-efficacy were also reported to achieve effectiveness as a result of the SBAR-based simulation program. SBAR-based education can improve critical thinking in the process of presenting various clinical judgment grounds to students and finding the best decision and evidence to confirm the decision [41]. In addition, it can be expected to improve self-leadership by giving individuals the spontaneity and self-direction necessary to judge, act, and perform work in a desirable way [21]. Furthermore, positive self-leadership can lead to self-confidence and self-efficacy in clinical performance. In previous studies, SBAR education and implementation positively improved patient safety competencies [21, 22]. Repeated use of SBAR helps to structure what to observe, what information to collect, and in what order to deliver content to alert the doctor; such structured information can enable nurses to make quick judgments and actions in urgent situations [22]. Therefore, the use of standardized communication tools facilitates proficient performance of nursing students and ultimately improves patient safety competency [22].

The interventions included in the analysis consisted of orientation, pre-briefing, role-play simulation, debriefing, or discussion. In 8 studies, pre-briefing was performed for more than 60 min. Pre-briefing may comprise several activities that include planning, using facilitation strategies, and transferring information. For novice nursing students who do not have experience or practice in thinking like a nurse or with the processes of reflection [42], a structured pre-briefing activity could support metacognition or critical thinking [43]. Indeed, theory-based, structured pre-briefing can impact nursing students’ clinical judgment, perceptions of pre-briefing, and competency performance and may enhance meaningful simulation learning [43]. Simulations consisted of role-playing or self-assertive training. Role performance simulation, including SBAR before graduation, helps new nurses improve their communication and information organization skills and the reliability of information delivery. In addition, assertive training has a positive effect on enhancing communication confidence and interpersonal relationships. When providing debriefing and discussion, it is adequate to avoid lecture-type methods and to receive feedback after directly observing one’s performance [44]. It is necessary to strengthen communication skills based on self-reflection and group reflection.

### Limitations

The program implemented in the literature included in this study confirmed the effectiveness of simulation education using SBAR. However, because there were differences in the intervention period, measurement methods, and intervention components of the programs, it was difficult to compare and analyze the effects in an integrated manner. In addition, programs implemented in the literature do not incorporate surveillance or other monitoring of fidelity to SBAR. This could potentially limit the effectiveness of simulation training using SBAR. Another limitation is the presence of additional interventions alongside SBAR in some included studies, which may have influenced the observed outcomes and made it difficult to isolate the specific impact of SBAR-based simulation training. Finally, additional studies reporting low fidelity or no improvement in communication clarity may not have been published; therefore, there is also a limitation due to publication bias.

### Implications for practice and future research

Simulation approaches in nursing education are now being proposed as a new pedagogical method to complement or replace clinical practice. The findings of the current study suggest that SBAR-based simulation programs have positive effects on nursing students’ capabilities for practice, with satisfaction and intense concentration in the provided situation. In future research, standardized and validated interventions for SBAR training should be researched for effectiveness during nursing education. Another potential research study would be to identify the effects of different simulation methodologies, such as web-based, high fidelity, and virtual simulations.

## Conclusion

This review provides a comprehensive update of the literature on the effectiveness of SBAR-based nursing simulation programs for nursing students. Our findings indicate that such programs lead to enhanced communication clarity and other positive learning outcomes among nursing students. However, given the variability in program components and measurement methods, it is essential to continue exploring the specific effects of SBAR-based simulation programs on various learning outcomes. This will enable a deeper understanding of the most effective strategies for optimizing communication and other crucial skills in nursing education.

## Data Availability

All data generated during this study are included in this published article.

## Abbreviations

SBAR: situation, background, assessment, and recommendation
EPHPP: Effective Public Health Practice Project
PICO-SD: participants, intervention, comparison, outcomes, study design
CENTRAL: Cochrane Central Register of Controlled Trials
CINAHL: Cumulative Index to Nursing and Allied Health
RISS: Research Information Sharing Service
KISS: Korean Studies Information Service System
Kisti: Korea Institute of Science and Technology Information
MeSH: Medical Subject Headings
CP: clinical practicum
CCS: communication clarity scale
GICC: communication competence scale
KidSIM-TPS: KidSIM team performance
VAS: visual analogue scale
